# Supplementary figures and images for: Identification of Novel Genes Associated with Fish Skeletal Muscle Adaptation during Fasting and Refeeding Based on a Meta-Analysis
Source: Genes (Basel). 2022 Dec 16;13(12):2378. doi: 10.3390/genes13122378 (PMC9778430; doi:10.3390/genes13122378)

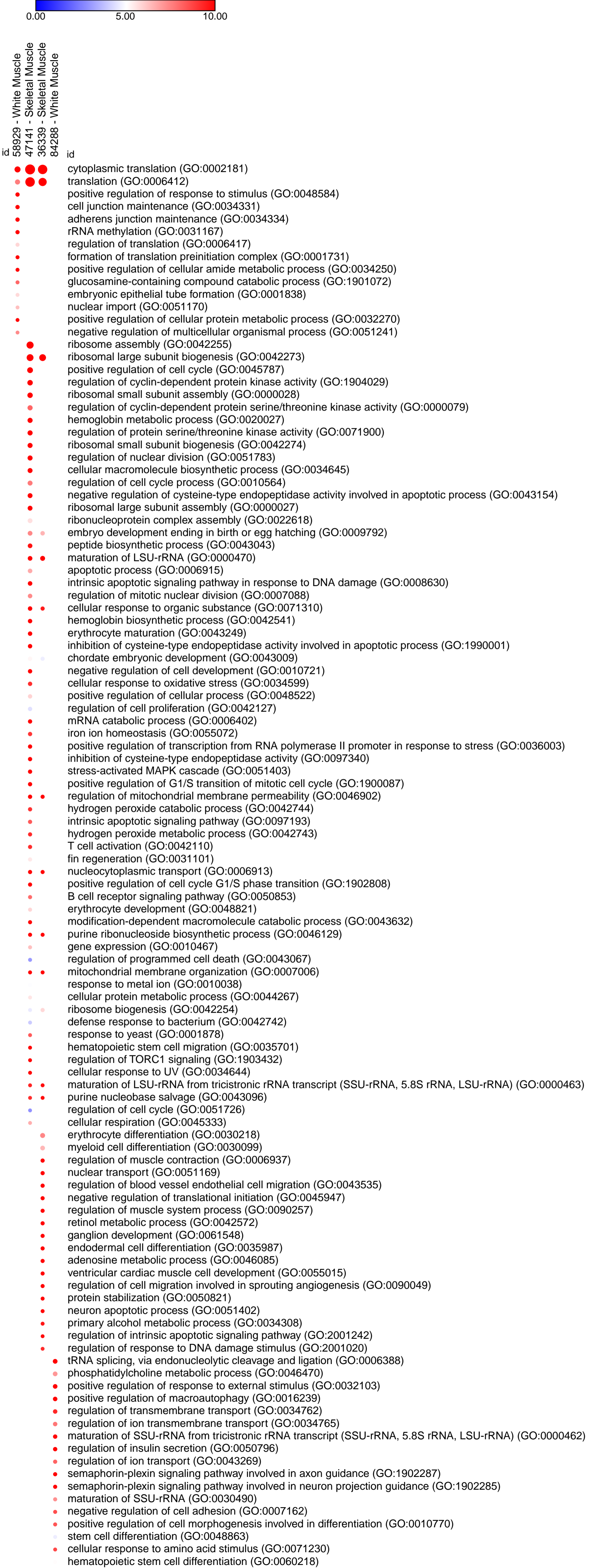

Supplement: Supplementary file 1 [file genes-13-02378-s001.zip › genes-2052642-supplementary/Figure S1.pdf]

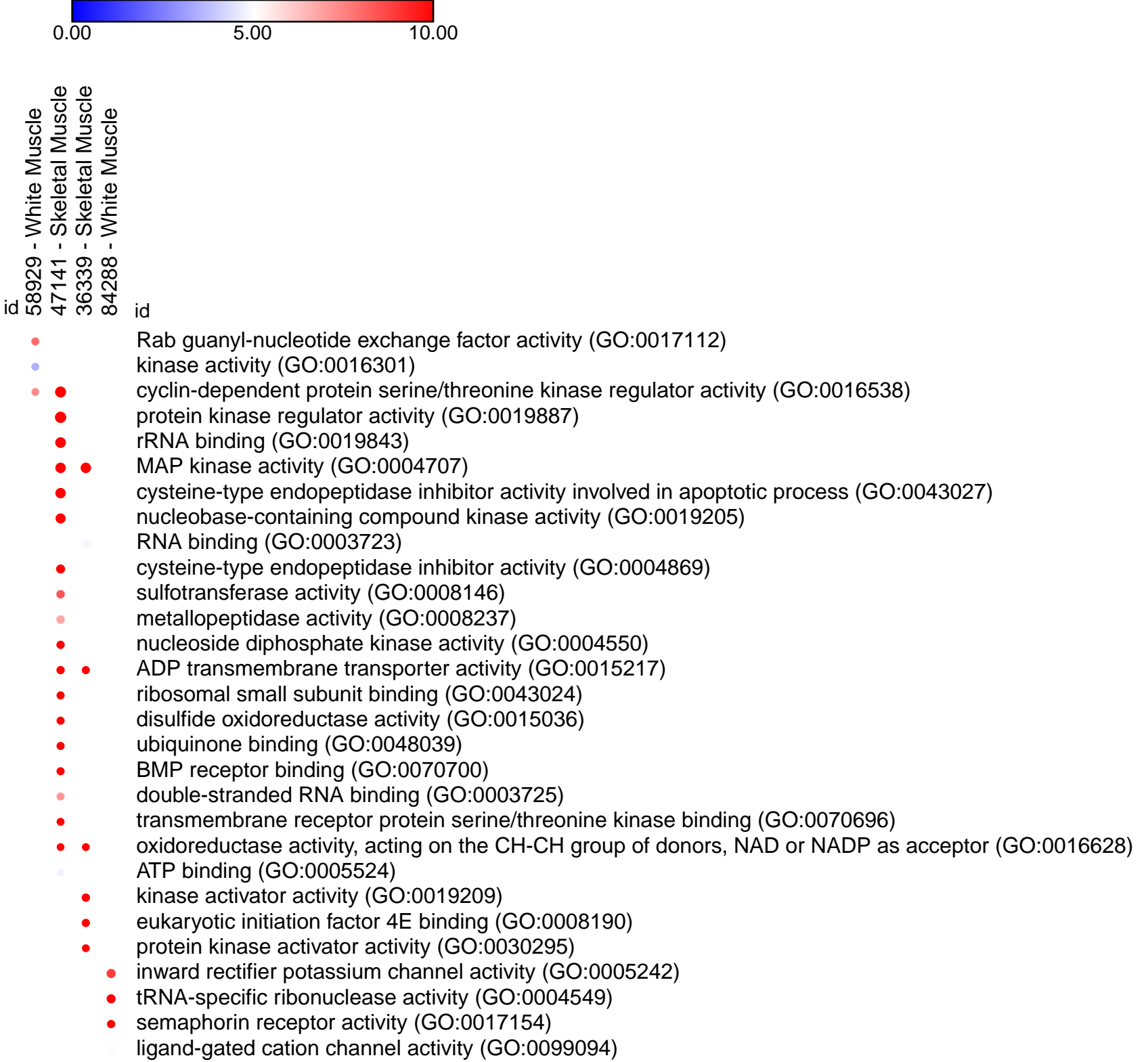

Supplement: Supplementary file 1 [file genes-13-02378-s001.zip › genes-2052642-supplementary/Figure S2.pdf]

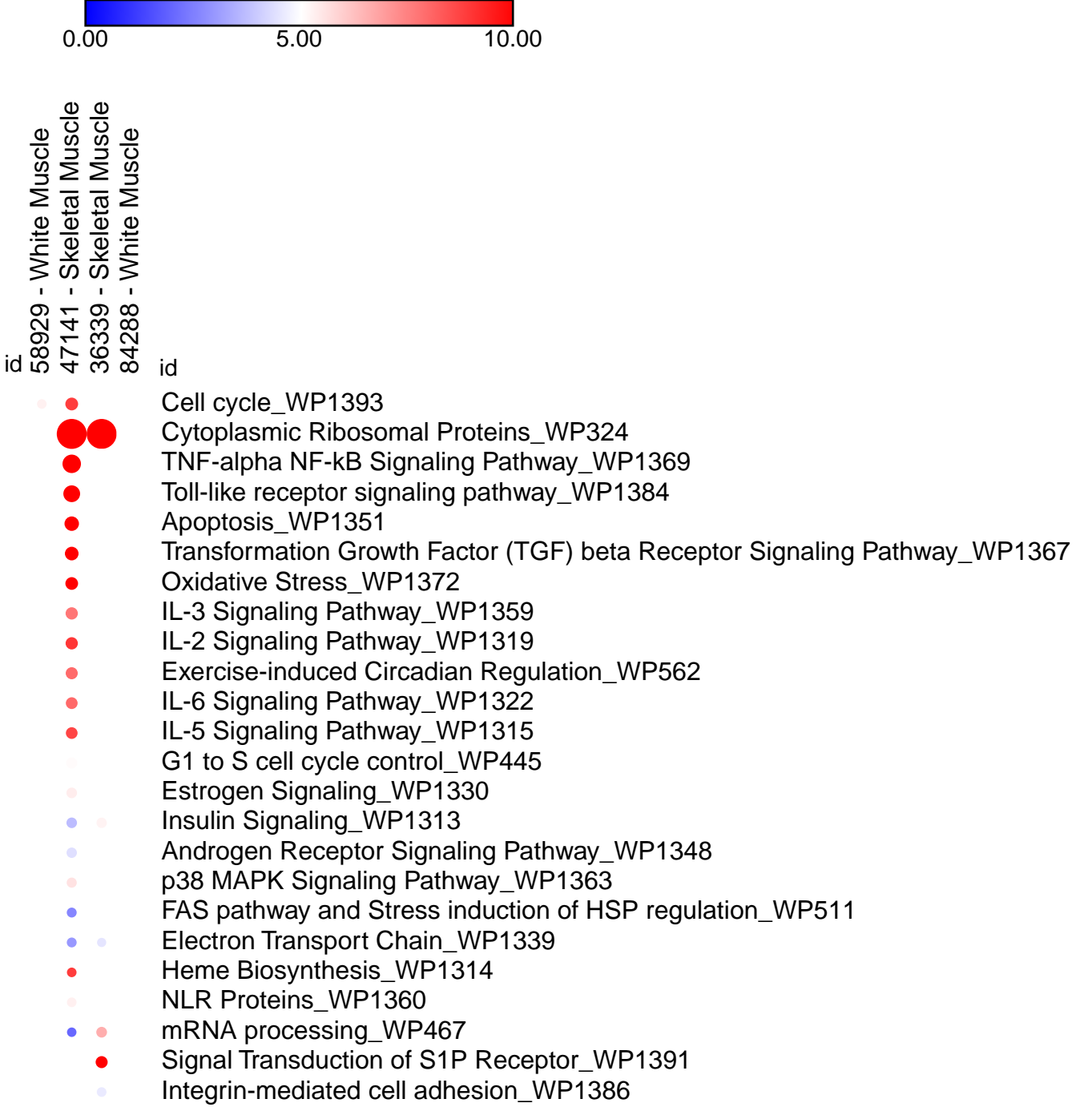

Supplement: Supplementary file 1 [file genes-13-02378-s001.zip › genes-2052642-supplementary/Figure S3.pdf]

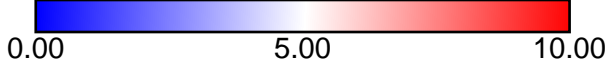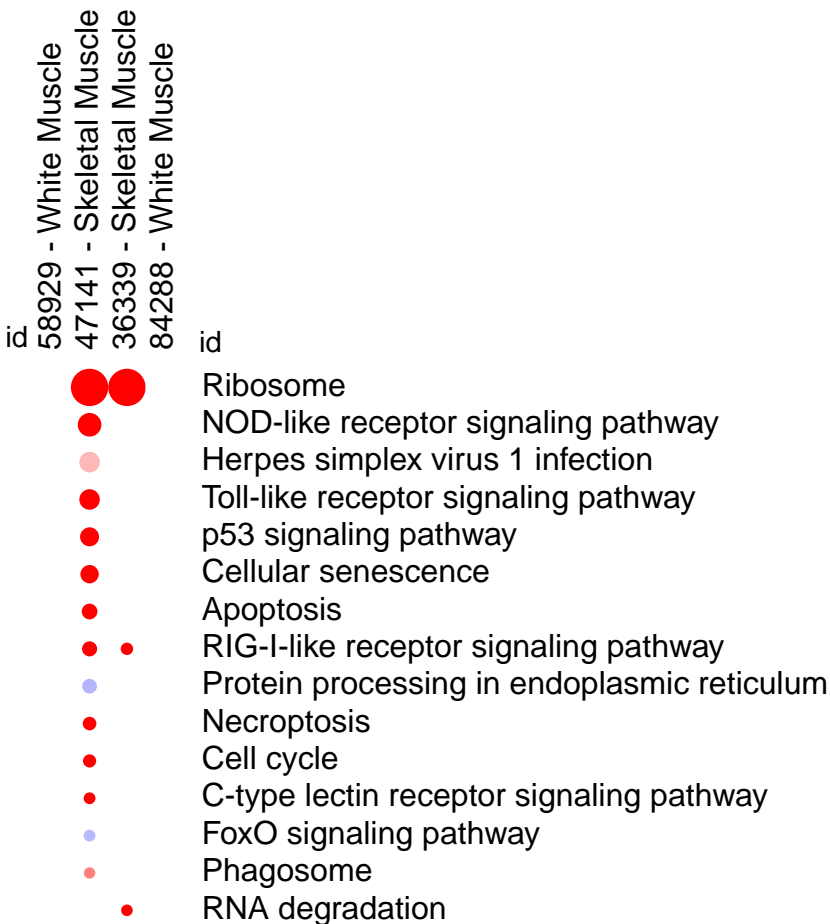

Supplement: Supplementary file 1 [file genes-13-02378-s001.zip › genes-2052642-supplementary/Figure S4.pdf]
